# Poisoned by Mercury from a Tooth Filling

**Published:** 1872-05

**Authors:** S. P. Cutler


### Dental Register.

Vol. XXVI.] MAY, 1872. No. 5.]

### COMMUNICATIONS.

### "Poisoned by Mercury from a Tooth Filling."

BY S. P. CUTLER, M. D., D. D. S.

In the January number of the Dental Register, 1872, I find the above heading, with details of the case, taken from a Nebraska paper. I need not go into all the details of the case again. The facts are these: "John T. Smith died from salivation, caused from having a tooth filled with amalgam. Dr. Sprague attended the case, and afterward called Drs. Davis and Buffon, all of whom agreed that he was suffering from the effects of mercury present in the amalgam used in filling one of his teeth. The filling had salivated the unfortunate man, and as the inside of his mouth, throat, and wind pipe swelled, respiration was hindered, and finally it ceased altogether."

Dr. Davis made the *post-mortem* examination, in presence of the Coroner and jury of inquest; opening the chest, taking out the lungs, and extracting the filled tooth. "No signs of any other disease were found, except that caused by the mercury, and it was made clear to the jury, by the doctor, that this caused his death."

S. W. Allen testified : "Mr. Smith had asked him to see a dentist for him; that Mr. Smith afterward told him that he had been to see Dr. Keef, who had filled the tooth; that he had filled it without killing the nerve, and that he, Smith, said that the dentist was intoxicated when he did the work."

The jury returned a verdict, that the deceased came to his death by suffocation, caused by inflammation of the glands, and infiltration of the tissues of the neck, producing closure of the trachia by pressure thereon; and we further believe that the above causes were brought about, by the action of mercury used in filling the second molar tooth of the right side of the lower jaw, by Dr. E. D. Keef, of Marysville, Kansas.

It does not appear in the testimony in the case, how long after the tooth was filled before death, nor how long after the tooth was filled before the trouble came on, nor whether or not the tooth itself began to cause trouble in, and about it, before symptoms of salivation came on; neither does it appear how large the filling was, or whether or not the filling came in contact with the gum, nor what portion of the tooth was decayed.

All these facts would be of importance in the investigation of so grave a case as this, where a life has been lost, and where censure is liable to fall on the dentist who filled the tooth, if not criminal prosecution. It does not appear in the investigation that mercury had not been taken by the patient before filling the tooth, for some other cause, and the system just at that time somewhat under its influence, and the salivation a simple coincidence only. It would appear from Smith's own statements about the dentist not killing the nerve in the tooth, that there must have been an exposure of the pulp. If so, the probability is, that the nerve died under the filling, producing all the horrible symptoms and consequences as detailed. The statement says: "It was known in town that he had been suffering for some days with a swelled face and neck, coming from a tooth, which Dr. Keeff, of Marysville, had lately filled, but his death was not thought possible."

If the medical gentleman in attendance had decided that the almalgam in the tooth caused the trouble, and the tooth itself, sore and tender, they should at once have removed the filling or the tooth; if the tooth was sore and painful, and the swelling first made its appearance about the tooth, it would be conclusive evidence of a dead or dying pulp under the filling, which in certain constitutions would be sufficien cause for all of the symptoms and consequences in the above detailed case. If the tooth filled was more affected than any of the other teeth, there is reasonable probability that a dead or dying pulp was the cause, and the same thing may have happened if os-artificial, gold or any thing else instead of amalgam had been used in filling. If the trouble was that of salivation alone, from the vapor of mercury during the process of hardening of the amalgam, the effects in the mouth would have been general, not local, nor confined to that tooth at all, as the action of the vapor would have to take place, first, through the lungs, then through the circulation, and localising itself afterwards; as the amalgam itself is not susceptible of producing any specific mercurial action, as no sensible change takes place in a filling in a tooth for a considerable length of time even then only a slight darkening the result of an oxide of silver, not of mercury, which is an insoluble, innocuous oxide, and perfectly harmless anywhere in the body. The acids of the mouth are too weak to produce salts of the materials of amalgam. The insignificant amount of evaporation of mercury in one filling could not amount to much. According to my experiments on amalgam, twenty-four grains of the fillings unite with fifteen grains of mercury, and lose two grains only by evaporation on drying; this amount of amalgam would more than make any common sized filling; half that amount would be largely over average size of amalgam fillings; the evaporation of mercury would only amount to about one grain or less, all told. I am fully convinced that this amount of vapor of murcury inhaled into the lungs, and absorbed by the blood, would not, under any circumstances, of itself produce salivation.

It is a well-known fact that salivation from mercury is a constitutional action, and that the "ptyalism" is only a local manifestation of the systemic effect, the liver being the organ primarily acted upon, and the salivary effect, a sympathetic, or reflex action. I have been alluding to the variety of mercurial action where there has been profuse ptyalism, or flow of salivary secretion, generally accompanied with periostitis, more especially of the lower jaws, frequently accompanied with excessive loosening of the teeth; in some instances they may be picked out with the thumb and fingers. In such cases, if there be dead or diseased teeth, or fangs, these are inflamed around them to a greater extent than healthy ones.

The swelling in such cases is very slight sometimes, at other times very considerable, accompanied with very distressing pain, in some cases almost intolerable. There is another variety of mercurialization, improperly termed, dry salivation, unaccompanied with ptyalism. In such cases there is a want of proper action of the liver, the secretions of bile not flowing out readily from the liver, that organ being under the influence of the mineral, but not to a sufficient extent to bring it to the active secreting point; in consequence, the sympathetic action on the glands of the mouth is a peculiar dry inflammation, which is always accompanied with more or less swelling of a malignant character, frequently ending in extensive sloughing of soft parts.

In this variety there is always more danger of suffocation from the extension of swelling to the throat than in the other, I have seen this variety cured by additional doses of mercury, on the principle of "*similia similibus curantur*," by carrying the action of the medicine to the secreting point, which exhausts itself from flow of saliva in time.

We are not told which variety of mercurial action was brought about by this amalgam filling. Pure quicksilver swallowed will not salivate, although certain portions must undergo vaporization while in the body, as the metal sends off vapors at all temperatures above sixty-six degrees.

Daguerreotypists used to keep a bath of mercury, always evaporating in their dark closets, and were necessarily in there a good deal, where they were constantly inhaling the fumes; and I never heard of one being salivated from that cause, and I have often asked them the question. It is a well-known fact that workers in quick silver do often suffer, and die in a few years, from constantly inhaling the fumes; this is a slow and insidious effect, not felt at first, though not direct salivation; the hair and nails frequently falling off in the advanced stages.

It seems to arrest nutrition, and produce constiutional waste, and exhaustion, and finally death.

Even admitting that this man did lose his life by the injudicious use of amalgam, *which I do not believe*, does it argue that amalgam should never be used under any circumstances? I have known many a life sacrificed by the use of mercurial drugs; does this prevent doctors from using it extensively? By no means. It should be a caution against the abuse of the mineral.

Why not discard rubber plates altogether, because "some dentists" say rubber always produces "sore mouths," when rubber is worn? (which I do not believe.)

Because chloroform causes death in some cases, is it a good reason for discarding it altogether? when lives are often lost from the shocks in formidable surgical operations, and are saved by the use of the drug? Certainly not.

The proper plan is to strike a balance between the good and bad effects of any agent, and decide in accordance with facts in the premises.

In the fall of 1862, I recollect a very serious case of swelling of the throat and neck, to such an extent that the life of the patient was almost despaired of for several days, in consequence of temporarily stopping the right second molar with gutta percha, which was followed with immediate swelling, so much so, that the mouth could not be sufficiently opened to remove the filling or tooth; in consequence, the case had to be treated with tooth and filling remaining in. Abscess was found and was lanced, followed by a copious discharge of pus, which also broke on the outside, and continued to discharge profusely for over two weeks, and subsequently indeed in a fistulous opening, which continued several years, until the removal of the tooth.

The inflammation in this case, at first, was erysipelas in character, accompanied with copious ptyalism, very similar to mercurialization, though I am not aware that the patient had taken mercury any time very recently before the occurrence.

The tooth was not worked at to any extent, when filled, and any other kind of stopping would have had the same result, as the patient took a severe cold just at that time.

Without the closest treatment aud attention, I believe this patient would have died from suffocation in consequence of the gutta percha filling.

It seems to me that the decision of the doctors and inquisitors was *a priori* descision, without a proper knowledge of all the facts in the case. Why was there an inquest held at all in in the case, unless the attending doctors wished to shirk responsibility, and cast the blame on the unfortunate dentist concerned? I again repeat, that if the tooth was the primary seat of trouble, the tooth should at once have been removed, which would, *no doubt in my mind*, have relieved the whole difficulty, and saved the life of the man.

